# 2385: An education program for engineering students collaborating with clinician scientists to address priority hospital patient safety problems using an ethnographic research approach

**DOI:** 10.1017/cts.2017.177

**Published:** 2018-05-10

**Authors:** Laura Camarata, Stephen P. Juraschek, Pamela Sheff, Peter A. Doyle, Robert M. Graham, John M. Adamovich, Lori A. Paine, Edgar R. Miller III

## Abstract

OBJECTIVES/SPECIFIC AIMS: Enhancing Patient Safety for hospitalized patients is a priority for healthcare facilities, providers, and federal funding agencies. Multidisciplinary partnerships in clinical and translational research better defines the scope of complex patient-safety issues, and is part of more effectively developing interventions. The discipline represented by engineering-trained partners brings valuable perspective to patient safety problems through their training background in human factors and systems analysis. The objective of this education program was to create and implement a collaboration between engineering students and clinical providers. Through the Johns Hopkins Institute for Clinical and Translational Research, a multidisciplinary partnership was created, to identify contributing factors, and suggest novel solutions, to key patient safety problems using an ethnographic research approach. METHODS/STUDY POPULATION: The collaboration was formed between the following Johns Hopkins (JH) groups: (1) The Institute for Clinical and Translational Research (ICTR), (2) The Armstrong Institute for Patient Safety, (3) The JH Hospital Clinical Engineering Services, (4) The Homecare Group, (5) The Masters of Science in Engineering Management Program at the Whiting School of Engineering, and (6) The JH Hospital Risk Management. All 6 provided representation to contribute to the planning, structure, and implementation of the project. The initial cohort was 24 masters students enrolled in the JHU Whiting School of engineering, and included 46% men, 54% women, and 75% international students. Students were placed in teams of 2–3 to work on 9 distinct patient safety concerns, as provided by the Armstrong Institute as priority. Potential clinical hosts from the appropriate clinical departments were vetted for feasibility and scope before students were assigned to them. Students and clinical hosts were oriented to the process. The students then spent 3–6 hours a week, for 7 weeks, observing and interacting with patients and health professionals at their specific clinical sites, conducting ethnographic research under the guidance of their hosts. Ethnographic research is the systematic investigation of a culture or system; in our application, teams were looking at the environment, culture, and its contributing factors, with respect to patient safety issues. Teams made observations, then formulated hypothesis and collected data relevant to what systems factors may be contributing to the patient safety issue. Following data collection and analyses, teams made recommendations for culture and/or systems shifts that could impact change and improve patient safety. Ethnography research process training is a tenet of the training undertaken by all Masters of Science in Engineering Management Students. RESULTS/ANTICIPATED RESULTS: At the end of the 7-week project, each team generated a comprehensive report suggesting potential solutions for each problem, and gave presentations on their findings to their peers, clinical hosts, and JHU steering committee representatives. Requirements on the student side included a midterm, final presentation, and report. Both students and site leaders submitted mid- and final program evaluations. Based on follow-up survey data, 71% of students said that the course may impact their career choice, 57% said the collaboration changed the way they viewed themselves, and 28% elected to continue working or were planning to work with their site in some fashion after the course ended. Nearly 60% of students believed additional funding or resources would benefit the course and 71% thought they would benefit from more or similar experiences with their clinical partners. Furthermore, 85% wanted to see the course expanded. Of the clinical hosts, 71% said that students added value, 86% believed students changed their perspective on their problem, unveiled new areas of investigation, and improved or likely would improve patient safety in their department. Seventy-one percent of hosts were actively acting on the students’ findings, and over 86% shared findings with their colleagues. Following the 7-week program, 2 teams also presented their findings to committees within the hospital departments, 2 patient-safety projects are being continued with engineering teams, and 2 new collaborative projects have been initiated. Based on the popularity of this program with the students, hosts, and teaching faculty, this will be implemented within the engineering curriculum for a second time next year. Additional outcomes data collection are currently ongoing, and we plan to continue to monitor and analyze results. DISCUSSION/SIGNIFICANCE OF IMPACT: In its first year our engineering collaboration exceeded expectations. Engineering students and clinical providers successfully worked toward tangible solutions that were directly applicable to patient care. Furthermore, interactions were both personally and professionally beneficial for students while simultaneously adding value to clinical hosts. Beyond the collaboration, this initiative allowed for secondary connections between engineers and clinicians that are also have great potential for resulting in translational innovation. Despite the overwhelming success of this project, it highlighted the need for increased resources for sustainability. Our pilot highlighted a role for funding with regards to: (1) students in the execution of their projects (eg, transportation to sites, prototype materials); (2) clinical hosts, particularly protecting time to interact with and lead student teams; (3) the Armstrong Institute—to aid the identification and prioritization of high impact, patient safety projects; and (4) the ICTR for staff to facilitate placements, student orientation to the hospital setting, and program execution and maintenance. Ultimately, this collaboration addressed an unmet need for the clinical providers as well as the engineering students: thus, all partners agree that (1) the impact of this pilot would be greatly magnified by more time, longer duration, and additional resources; and (2) this collaboration could provide a useful model for approaching other complex health care problems. In terms of larger and longer-term impact, engaging engineers at the training level together with clinicians provides early exposure, and could potentially prime them to continue collaborations with clinical and translational science, across their careers.

Student Research Assistant Acknowledgements: The authors thank Manik Arora, Alexandra Morani, and Thomas Cornish -- Johns Hopkins University.

